# Metabolic complications and treatment of perinatally HIV-infected children and adolescents

**DOI:** 10.7448/IAS.16.1.18600

**Published:** 2013-06-18

**Authors:** Linda Barlow-Mosha, Allison Ross Eckard, Grace A McComsey, Philippa M Musoke

**Affiliations:** 1 Makerere University-Johns Hopkins University Research Collaboration, Kampala, Uganda; 2 Department of Pediatrics, Division of Infectious Diseases, Emory University School of Medicine, Atlanta, GA, USA; 3 Department of Pediatric Infectious Diseases and Rheumatology, Case Western Reserve University, Cleveland, OH, USA; 4 Department of Paediatrics and Child Health, School of Medicine, College of health Sciences, Makerere University, Kampala, Uganda

**Keywords:** children, adolescents, HIV, antiretroviral therapy, metabolic complications, cardiovascular disease

## Abstract

The benefits of long-term antiretroviral therapy (ART) are recognized all over the world with infected children maturing into adults and HIV infection becoming a chronic illness. However, the improved survival is associated with serious metabolic complications, including lipodystrophy (LD), dyslipidemia, insulin resistance, lactic acidosis and bone loss. In addition, the dyslipidemia mainly seen with protease inhibitors may increase the risk of cardiovascular disease in adulthood and potentially in children as they mature into adults. Nucleoside reverse transcriptase inhibitors, particularly stavudine, zidovudine and didanosine are linked to development of LD and lactic acidosis. Perinatally infected children initiate ART early in life; they require lifelong therapy with multiple drug regimens leading to varying toxicities, all potentially impacting their quality of life. LD has a significant impact on the mental health of older children and adolescents leading to poor self-image, depression and subsequent poor adherence to therapy. Reduced bone mineral density (BMD) is reported in both adults and children on ART with the potential for children to develop more serious bone complications than adults due to their rapid growth spurts and puberty. The role of vitamin D in HIV-associated osteopenia and osteoporosis is not clear and needs further study. Most resource-limited settings are unable to monitor lipid profiles or BMD, exposing infected children and adolescents to on-going toxicities with unclear long-term consequences. Improved interventions are urgently needed to prevent and manage these metabolic complications. Longitudinal cohort studies in this area should remain a priority, particularly in resource-limited settings where the majority of infected children reside.

## Introduction

Potent antiretroviral therapy (ART) has significantly reduced the morbidity and mortality of HIV-infected adults [1] and children [1–3]. The long-term benefits of ART are associated with metabolic complications, including lipodystrophy (LD), dyslipidemias, lactic acidosis, glucose intolerance, osteopenia and osteoporosis [4–9]. The current World Health Organization (WHO) ART guidelines recommend the initiation of paediatric treatment early in life leading to prolonged ART exposure through various stages of growth and development, treatment with multiple drug regimens and a higher risk for metabolic complications [8–10].

Metabolic complications of ART are well-documented in HIV-infected adults and children, although paediatric cohort studies are limited [4,8]. The nucleoside reverse transcriptase inhibitors (NRTIs), stavudine (d4T), zidovudine (AZT) and didanosine (ddI) are closely linked to LD and lactic acidosis [11]. Protease inhibitors (PI) have consistently been associated with dyslipidemias (increased cholesterol and triglycerides) in children which may increase the risk of cardiovascular disease (CVD) in adulthood [4,5,8,12]. A recent study has reported vitamin D deficiency in youth, which may occur as a complication of ART and result in bone demineralization [13]. Reduced bone mineral density (BMD) has been well described in HIV-infected adults and more recently similar bone loss has been reported in children on ART [14,15]. The combination of severe malnutrition and concurrent micronutrient deficiencies in children initiating ART in resource-limited settings may lead to further reductions in BMD in these populations [16]. The aim of this review is to discuss the epidemiology, clinical presentation and management of metabolic complications of perinatally HIV-infected children and adolescents on ART.

## Lipodystrophy syndrome

LD syndrome is increasingly being recognized as a common complication among HIV-infected children and may be associated with hyperlipidemia and insulin resistance (IR) [17]. Body fat maldistribution is especially problematic for adolescent patients who are generally sensitive to their body image, vulnerable to depression, and prone to antiretroviral non-adherence [17]. These body changes often lead to stigmatization, which in turn may lead to poor adherence and ultimately to treatment failure. LD syndrome encompasses changes in regional fat distribution manifesting as lipoatrophy (LA), with or without central adiposity (lipohypertrophy-LH) [7] and is frequently associated with abnormalities in lipid regulation and glucose homeostasis. Children affected with LD exhibit different patterns and severity of fat maldistribution; however, similar to adult subjects, LA is more specific for HIV infection and constitutes a key component of LD [7]. Aurpibul *et al*. noted that LH and LA often occur independent of one another [18]. Dyslipidemias can occur in the absence of LA and LH [7,19,20]. As more HIV-infected children receive life-long ART, the long-term consequences of LD and the associated dyslipidemias and IR, may increase their lifetime risk of CVD. However, long-term data for children as they progress into adolescence and young adulthood are lacking.

### Epidemiology

The prevalence of LD ranges from 1 to 57% among HIV-infected children [5,20,21] and from 2 to 84% among HIV-infected adults [7]. In Europe, a recently completed cross-sectional analysis among HIV-infected children (*n*=426) aged 2–18 years with a median duration of 5.2 years on ART, reported a prevalence of 57% for LD [20]. A prospective longitudinal study among HIV-infected children in Thailand reported a prevalence of LD of 9, 47, and 65% at 48, 96, and 144 weeks, respectively, after non-nucleoside reverse transcriptase inhibitor (NNRTI) based ART [18]. In two sub-Saharan African studies, the prevalence of LD ranged from 27 to 30% among children aged 1–18 years [21,22]. Both these studies found that older children and the use of d4T are significant risk factors for LA. The prevalence of LD in children varies by geographic regions depending on the use of PI-based regimens, stavudine-containing therapy, and the availability and duration of ART. In addition, differences in methods used to determine and define LD in these studies complicate the estimation of true prevalence of LA and LH.

### Aetiology

Although the precise mechanisms of LD are not well understood, several hypotheses have been proposed (Table 1). The pathogenesis of ART-associated LA and LH differs; it is complex and multifactorial, including direct effects on lipid metabolism, genetic polymorphisms, mitochondrial and adipocyte cell function [33,34]. Mitochondrial DNA is affected by both HIV infection and NRTI therapy [27,28]. Exposure to NRTIs, including d4T and zidovudine (AZT), and to a lesser degree to PIs, has been implicated in the development of LA/LH [11,35–38]. Mitochondrial dysfunction could lead to decreased ATP, decreased lipogenesis and increased pro-apoptotic mediators, which result in fat apoptosis [23,29]. Puberty has been identified as a time when LD is most likely to develop [7,22]. There is no consensus about whether females are more likely to have LD compared to males with some studies reporting higher prevalence in females and others higher in males [5,18,22]. A study by Resino *et al*. has also shown a higher prevalence of LD among HIV-infected children with rapid immunologic recovery [39].

**Table 1:** Potential aetiology of lipodystrophy syndrome complication

	Mechanism	Available data
Lipoatrophy	Mitochondrial toxicity	NRTIs inhibit mitochondrial DNA (mtDNA) polymerase gamma, leading to mtDNA depletion, respiratory chain dysfunction, and reduced energy production [23–26]. However, the function of mitochondrial DNA is affected by both HIV infection and NRTI [27,28]. Mitochondrial dysfunction could lead to decreased ATP, decrease lipogeneis, and increased pro-apoptotic mediators, which result in fat apoptosis [23,29].
Lipoatrophy/ lipohypertrophy	Effect of protease inhibitors	PIs have a high affinity for a site of HIV-1 protease, which shares a sequence homology with 2 proteins involved in lipid metabolism, cytoplasmic retinoic acid–binding protein type 1 (CRABP-1), and low-density lipoprotein receptor–related protein (LDLR-RP) [30]. Inhibition of CRABP-1 impairs the production of retinoic acid, which leads to decreased fat storage and adipocyte apoptosis. Subsequently lipids are released into the circulation [30].
Dyslipidemia	Effect of protease inhibitors	Inhibition of LDLR-RP results in hyperlipidemia due to the failure of hepatocytes and endothelial cells to removal of triglycerides from the circulation [30].
Glucose homeostasis	Inhibition of GLUT-4	Both PIs and NRTIs have also been associated with insulin resistance, through inhibition of muscular and adipocyte GLUT4 (insulin-regulated transmembrane glucose transporter), resulting in decrease glucose intake mediated by insulin in these tissues [31,32].

### Clinical presentation

There are three patterns of body fat maldistribution: (1) LA: with decrease subcutaneous fat in the face, limbs and/or buttocks; (2) Lipohypertorphy: with accumulation of fat in the upper chest, abdomen, breast and/or dorsocervial region; (3) mixed/combined pattern with both LA and LH. Although LA is the most characteristic fat redistribution in adults, there is no consensus for children [7]. A study among Thai children found a 46% prevalence of central LH, 20% peripheral LA, and 34% combined pattern after 144 weeks of NNRTI-based ART [18]. However, a cross-sectional study in Uganda reported that LA with facial wasting was the most common body shape change among children with fat distribution after a median duration of 3.8 years on ART [22]. A recent study among European children found that LA occurred in 28% (* n*=117), and LH in 27% (*n*=115), most commonly in the face and trunk, respectively [20]. In multivariable analysis, white ethnicity, body mass index (BMI) and exposure to lopinavir/ritonavir (LPV/r) and NNRTIs were each associated with increased risk of LD (*p*<0.05). White ethnicity, history of CDC-defined disease and d4T were associated with risk of LA (*p*<0.05) [20].

#### Dyslipdemia

Dyslipidemias are a common component of ART-associated LD. However, low levels of high-density lipoprotein cholesterol (HDL), low levels of low-density lipoprotein cholesterol (LDL-C) and elevated triglycerides have been associated with HIV in adults [40,41]. The definition of hypercholesterolemia and hypertriglyceridemia varies among studies. Several guidelines to determine cut-off points for abnormal lipid levels for children and adolescents have been published, including the National Heart, Lung and Blood Institute (NHLBI)-released Expert Panel on Integrated Guidelines for Cardiovascular Health and Risk Reduction in Children and Adolescents, November 2011, which was endorsed by the American Academy of Pediatrics [42–44].

Taylor *et al*. reported that receiving PI therapy in the age range of 10–15 years and sustained control of viremia were associated with the development of fat redistribution and dyslipidemia [35]. All PIs are associated with elevated TG, LDL-C and total cholesterol levels [45]. Among the NRTIs, d4T is associated with increased levels of TC, LDL-C and TG [46]. Apradi *et al*. compared metabolic abnormalities in HIV-infected children on LPV/r to nevirapine (NVP)-based ART and found significantly higher LDL-C and TG levels among children who remained on LPV/r [12]. While the long-term CVD risk for HIV-infected children on ART is unknown, the observed elevations in cholesterol levels are similar to those seen in patients heterozygous for familial hypercholesterolemia and, therefore, may confer a similar risk for premature atherosclerotic disease [47].

#### Insulin resistance

Insulin resistance is characterized by the decreased ability of insulin to stimulate the use of glucose by muscles and adipose tissue leading to increased production of pancreatic insulin. A variety of disorders of glucose metabolism have been associated with HIV infection and ART, including impaired glucose tolerance, impaired fasting glucose and type 2 diabetes mellitus (DM). Unlike adults, disturbances in glucose homeostasis are relatively uncommon in HIV-infected children. Studies have shown differing results on the association of glucose homeostasis with PIs and LD syndrome [48,49]. Impaired glucose homeostasis has been reported among 8–35% of HIV-infected children [31]. However, no differences were detected in fasting serum insulin, proinsulin, C-peptide, insulin:glucose ratio or Homeostasis Model of Assessment (HOMA-IR) between PI-treated and PI-naïve children [48,50–52]. Normal fasting glucose level and glucose tolerance tests have been reported among children with LD [18,22,53,54]; however in this setting, high fasting insulin concentrations were found primarily among children with LH and inconsistently with LA [53,54]. However, prolonged exposure to high insulin levels may increase their risk of type 2 DM. There are limited longitudinal data on IR among HIV-infected children on ART but some reports document an increased prevalence over time [55,56].

### Diagnosis

#### Fat distribution

A variety of techniques can be used to diagnose LD (Table 2); however, clinical presentation remains the most commonly used method, especially in resource-limited settings. Systematic objective measurements are required to detect abnormalities of fat distribution unless LD is severe enough to be recognized by the physician or caretaker. Anthropometric measurements are an inexpensive way to measure fat distribution, but they require significant standardization and experience and only measure subcutaneous fat [7]. While some studies have used dual-energy X-ray absorptiometry (DXA) to assess fat distribution in HIV-infected children [54,61,62], the cost and availability in a resource-limited setting are prohibitive.

**Table 2:** Diagnosis of lipodystrophy syndrome

Complication	Technique	Comment
Fat distribution (lipoatrophy and lipohypertrophy)	Anthropometric measurements (waist-to-hip ratio, skinfolds, limb circumferences)	An inexpensive way to measure fat distribution but, they require significant standardization and experience, and only measure subcutaneous fat [7].
	Bioelectrical impedance (BIA)	Measures lean body mass and total body fat but not regional fat distribution [57].
	Dual-energy X-ray absorptiometry (DXA)	Measures regional fat distribution (except facial fat) and is ideal for longitudinal studies [58].
	Computed tomography (CT) and magnetic resonance imaging (MRI)	Both discriminate well between subcutaneous fat and visceral fat, however both are expensive and may require sedation for young children [7].
Dyslipidemia	Fasting and non-fasting lipid levels	The cut off points for abnormal lipid levels were defined as follows: total cholesterol ≥200 mg/dl, low density lipoprotein cholesterol (LDL) ≥130 mg/dl, triglycerides (TG) ≥100 mg/dl in children 0–9 years, and TG ≥130 mg/dl in adolescents 10–19 years of age [43]. If lipid abnormalities are found then secondary causes should also be assessed such as obesity, hypothyroidism, and diabetes mellitus.
Glucose homeostasis	Hyperinuslinaemic euglycemic clamp	This is the gold standard to assess insulin, however it is an expensive and labour intensive technique, primarily suitable for research alone [59].
	Fasting glucose, fasting insulin, C-peptide, and oral glucose tolerance tests (OGTT)	Fasting insulin and glucose levels and indices derived from the OGTT correlate well with hyperinsulinaemic euglycemic clamp both in adults and paediatrics [59,60].
	Homeostatic model assessment (HOMA-IR), the fasting glucose:insulin ratio and the quantitative insulin sensitivity check index (QUICKI)	The most frequently used in clinical investigations are fasting insulin resistance (IR) indices [50,59].

#### Dyslipidemia

Lipid profiles should be obtained from all children prior to the initiation of ART. Thereafter, they should be repeated every 6–12 months. In resource-limited settings where facilities to measure blood lipid levels are not available, the collection of dried blood spots and transfer to reference laboratories should be utilized [16,63]. Guidelines for screening have been published by the National Cholesterol Education Program Expert Panel [49]. However, an updated classification was published by Jolliffe and Janssen with age- and gender-specific lipid thresholds for adolescents aged 12–20 years [42].

#### Insulin resistance

A variety of methods have been used to diagnose IR, including the measurement of fasting glucose, fasting insulin, C-peptide, oral glucose tolerance tests (OGTT) and derivations of various indices generated from these values [7]. The gold standard to assess IR is the hyperinsulinaemic euglycemic clamp [59]. Fasting insulin and glucose levels and indices derived from the OGTT correlate well with hyperinsulinaemic euglycemic clamp both in adults and paediatrics [59,60].

### Management

#### Fat distribution

Switching the suspected offending antiretroviral agent has been the most common strategy to manage fat maldistribution in LD. In cases of LA, avoidance of d4T, ddI, and to a lesser extent AZT, are recommended and substitution with either abacavir (ABC) or tenofovir (TDF). Few studies using switch strategies for LA have been conducted in children. Vigano *et al*. reported on changes in body composition in a study where a simultaneous switch was made from d4T to TDF and from a PI to efavirenz (EFV) among 24 virologically suppressed HIV-infected children with LA, aged 5–17 years [64]. This prospective study compared body composition after the switch to that of healthy controls using DXA. Restoration of physiologic fat accrual and no further progression of LA was reported 96 weeks after replacement of d4T with TDF and a PI with EFV [64]. However, Gonzalez-Tome *et al*. reported no significant changes in body fat composition after substitution of a PI with NVP [65]. Other investigational strategies have been identified to manage LD including the use of growth hormone (GH) and other drugs. Impaired GH has been correlated to visceral adiposity [66]. A study among adolescents reported visceral fat reduction with the use of recombinant GH [67]. However, patients may develop glucose intolerance as a result of GH therapy. Other potential treatments include metformin, thiazolidinediones, and testosterone [55], but results have been conflicting. Reconstructive surgery may be considered for adolescents with disfiguring fat maldistribution and psychological problems [68]. However, surgical management of LD is only efficacious with lipohypertrophy [69]. Various procedures in adults have recently been proposed for facial LA including polylactic acid injections, fat autotransplantation and silicone implants [70].

#### Dyslipidemia

The first step in management of dyslipidemias is lifestyle modification with a low-lipid diet and aerobic exercise. If the child is on a PI-based regimen then studies have shown that switching to a PI-sparing regimen or atazanavir (ATV) can reduce TC and TG levels [71]. McComsey *et al*. studied 17 children with viral suppression who were switched from a PI-containing regimen to EFV, with significant improvements in TC, LDL, TG and sustained viral suppression after 48 weeks [72]. Another prospective study which randomized 28 children to switch from PI to EFV and d4T to TDF at baseline (group 1) or 24 weeks (group 2) showed a significant improvement in lipid profiles at 48 weeks after substitution [73]. However, since both PIs and d4T were switched at the same time, it was difficult to attribute the improvement to a specific antiretroviral drug.

If there is inadequate response after 6–12 months of the initial intervention, then lipid-lowering drugs such as statins (pravastatin and atorvastatin) may be considered for children ≥8–10 years with LDL levels >190 mg/dl or >160 mg/dl with a family history of CVD [43]. There are limited data on the use of resins (bile acid sequestrants) and cholesterol-absorption blockers (Ezetimibe) in HIV-infected children; however, these drugs are Food and Drug Administration (FDA)-approved for use in children with familial hypercholesterolemia.

#### Insulin resistance

Lifestyle changes in diet and exercise are the first intervention to manage IR. If a PI is the suspected cause of insulin resistance, studies in adults and children have shown switching to a PI-sparing regimen or unboosted atazanavir could improve insulin sensitivity [50,56,74]. Vigano *et al*. conducted a four-year prospective study of PI-treated HIV-infected children which showed that a treatment switch to an NNRTI-based treatment was associated with an improvement in insulin sensitivity compared with the previous PI-based regimens [56]. However, if substitution fails then metformin can be used in children >10 years of age. Metformin has been shown to improve insulin sensitivity and BMI in non-diabetic obese adolescents with fasting hyperinsulinemia and a family history of type 2 DM [50]. However, metformin should be used with caution in children receiving NRTIs because of the rare complication of lactic acidosis. Other potential agents are thiazolindinediones (rosiglitazone, pioglitazone), which improve insulin sensitivity in HIV-infected adults with LD, but are not yet FDA-approved for children.

Preventive measures of LD should be incorporated in routine care, with active surveillance for fat maldistribution. In resource-limited settings, as the use of PIs (LPV/r) as first-line for children increases, monitoring of lipid levels and provision/availability of alternative antiretrovirals will become necessary and potentially lipid-lowering agents for severe hypercholesterolemia. More data are needed on the long-term outcome of HIV-infected children with early signs of IR and management in young children.

## Cardiovascular disease

HIV-infected adults have an increased risk of CVD compared to the general population [75,76]. Both abnormal lipoprotein profiles and increased inflammation have been demonstrated in multiple studies of HIV-infected children and adolescents [77–81]. Abnormal lipid profiles are also associated with inflammatory markers [81–83]. In addition, endothelial dysfunction, underlying vascular disease and arterial stiffness have been associated with heightened inflammation and/or immune activation in HIV-infected adults and children [84–88].

Because clinical cardiovascular events are expected to be of low prevalence, non-invasive techniques have been widely used as surrogates of CVD risk in both adults and children with HIV. Pulse wave velocity (PWV), which measures arterial stiffness, and carotid intima-media thickness (IMT) measured by ultrasound are two of the most well-accepted and robust methods to estimate subclinical arterial stiffness and vascular disease. Each of these tests is a powerful and independent predictor of CVD events in various populations, even after adjustment for traditional CVD risk factors [89–95].

A number of cross-sectional studies have also found increased carotid IMT in HIV-infected children and adolescents compared to healthy uninfected controls [77,96–98]. To date, one study has evaluated longitudinal carotid IMT data [83] and found that in both the HIV-infected and control groups, IMT decreased (*i.e*. improved) over the 48-week time period, with more pronounced changes among the HIV-infected group for both internal carotid artery (ICA) and common carotid artery (CCA) IMT. While higher CD4+ T-cell count and longer duration of ART may have contributed to the improvements seen, it is generally unknown what the natural course of carotid IMT is in this population. As Fernhall *et al*. [99] pointed out in a thorough review of the literature among healthy children, discrepancies among various studies may be due to the fact that IMT changes very little during childhood, and as it changes, so does arterial size and luminal diameter [100,101]. These complications likely make measuring carotid IMT longitudinally in children much more challenging and difficult to interpret than in adults, and thus may limit its use in this population. PWV has also been evaluated in HIV-infected children, but only in one cross-sectional study, which showed that HIV-infected subjects had worse PWV compared to healthy controls [102].

While there are limited data evaluating subclinical atherosclerosis among HIV-infected adolescents, the fact that they have abnormal lipoprotein profiles and increased inflammation suggest that they too are at an increased CVD risk like their adult counterparts. Given the additive risk associated with HIV infection, evaluating CVD risk in HIV-infected adolescents is of paramount importance as the number of long-term survivors of perinatally infected children and behaviourally infected adolescents is growing at a significant rate due to combination ART. In addition, assessing the effect of safe interventions on CVD risk aimed at decreasing inflammation should be one of the primary research goals in the coming years. The challenge in resource-limited settings is that most of the diagnostic tests for CVD are not accessible to most infected children. Therefore, simpler tests and interventions need to be evaluated and prevention strategies implemented.

## Lactic acidosis

Hyperlactatemia is a well-recognized complication of ART with the spectrum of disease ranging from mild to moderate asymptomatic hyperlactatemia to fulminant life-threatening lactic acidosis with lactate levels >5 mmol/L and hepatic steatosis [103]. The mechanism for severe lactic acidosis has been linked to NRTI inhibition of mitochondrial DNA (mtDNA) polymerases leading to mtDNA depletion. Stavudine and ddI have the greatest effect on mtDNA, with AZT, 3TC, TDF and ABC having less effect (in decreasing order). Chronic mitochondrial toxicity leads to mtDNA depletion and finally dysfunction with disturbance of oxidative phosphorylation and shifting of the pyruvate–lactate equilibrium to lactate [104]. The clinical presentation is non-specific, including asthenia, malaise, vomiting, abdominal pain, weight loss, tachypnoea, dyspnoea, and muscle weakness. The most common laboratory abnormalities include an increased anion gap, elevated transaminases, increased creatinine phosphokinase (CPK), lactate dehydrogenase deficiency (LDH), amylase and lipase [103].

Mild to moderate asymptomatic hyperlactatemia is frequently reported with an estimated prevalence of 15–30% in adults and 35–50% in children [105]. The incidence of severe lactic acidosis ranges from three to 10 episodes/1000 person-years on ART [106,107]. In children, mild to moderate asymptomatic hyperlactatemia has been described but severe lactic acidosis is rare [108,109]. A large cohort of 1422 children in South Africa reported a low rate of d4T toxicity requiring medication changes at 28.8/1000 years on treatment with only three cases of lactic acidosis [110]. The majority of medication substitutions were due to LD. The authors conclude that where there are limited drug options, d4T remains relatively safe. In contrast to adults, d4T has less toxicity in children, but the risk of LD remains, especially as children remain on disproportionately higher doses of d4T compared to adults [38]. Shah reported non-fatal lactic acidosis in two HIV-infected Indian children on a d4T based regimen for five and three years, respectively, when they presented with vomiting and diarrhoea [109]. Rey *et al*. reported a fatal case of lactic acidosis in a five-year old child on d4T and ddI [108] and Carter *et al*. reported a 10-year old male with severe lactic acidosis while on d4T, ddI and NVP [111]. These cases emphasize the increased risk of lactic acidosis with d4T alone or in combination with ddI.

Noguera *et al*. documented at least one measurement of hyperlactatemia over a 28-month period in 23 of the 80 children on ART (with the majority on a NRTI backbone). Fourteen of the 23 (61%) had asymptomatic hyperlactatemia [112]. None of the children had lactic acidosis. Hyperlactatemia in these children was associated with higher CD4 cell count and younger age at ART initiation [112]. Another study, a retrospective chart review of 127 children, with 104 on ART, identified 41 (32%) with asymptomatic hyperlactatemia (lactate >2 mmol/l), but none of the children developed severe lactic acidosis. The hyperlactatemia was associated with NRTIs and PIs regardless of treatment regimen and viral suppression [113]. In conclusion, most of the children with hyperlactatemia are asymptomatic and do not require a specific intervention.

Management of lactic acidosis requires a high index of suspicion and confirmation with measurement of a venous blood lactate level. If confirmed, then the offending NRTI, usually d4T and ddI alone or in combination, should be stopped and TDF or ABC substituted [114]. Anecdotal reports document the benefit of antioxidants including thiamine, riboflavin and L-carnitine, but there are no randomized-controlled trials. The prevention of hyperlactatemia requires the use of second generation NRTIs that have a lower capacity to inhibit DNA polymerase gamma [115]. However in cases of lactic acidosis, NRTI-sparing regimens are advisable.

## Bone disease

Multiple studies have demonstrated decreased BMD in HIV-infected adults with a 15 and 52% prevalence of osteoporosis and osteopenia, respectively [116–118]. This decreased BMD results in an increased risk of fractures in this population [119]. The effects of HIV and ART on bone health among HIV-infected children and adolescents may be even more detrimental than in adults. Most adolescents with perinatal HIV infection, for example, have been on ART for much of their lives, including through puberty which is a time of rapid growth and bone mineral accrual [120]. They will likely continue on ART for decades to come, potentially putting them at significant risk for osteoporosis and subsequent fractures later in life. Despite this, data on bone disease in this population remain sparse.

### Epidemiology

A number of studies have investigated the prevalence of low BMD in this population. Different criteria to define low BMD and diverse subject populations make it challenging to compare results among studies. However, most studies show that a quarter to half of subjects have low BMD, as defined by a Z-score of ≤−2 as per the 2007 International Society for Clinical Densitometry Pediatric Official Positions [15,121–126]. In most studies, these numbers are significantly lower than matched healthy adolescents [124,126–129].

In contrast to the aforementioned studies, a recent multi-centred, cross-sectional analysis of a relatively large cohort of perinatally infected adolescents showed not only a lower prevalence of low BMD (23 and 21% of HIV-infected subjects had a total body and lumbar spine sex- and age-adjusted BMD z-score <−1.0, respectively), but after adjusting the mean total body Z-scores for sex, race, pubertal maturity, height, weight, and BMI Z-score, there were no differences between the HIV-infected group and the HIV-exposed but uninfected group [130]. This study adjusted for many variables that are known to be altered by HIV infection and/or its therapy; thus, the results of this study should be interpreted with caution. In addition, the proportion of HIV-infected subjects with total body BMD Z-scores <−2.0 was significantly increased compared to controls (7% vs. 2%, *P*=0.019), with the HIV-infected subjects having double the expected rates compared to normal population distributions.

Moreover, in this study, most of their subjects had not yet entered their adolescent pubertal growth spurt. Adolescent years are crucial for bone health as they are associated with the greatest accumulation of bone mass, and attainment of 80% of peak bone mass occurs by 18–25 years of age [129,131–133]. Thus, this is a particularly vulnerable time, and any impairment of bone gain may impact lifelong bone health. For example, Jacobson *et al*. showed that HIV-infected adolescents, particularly boys, had lower BMD at the end of puberty compared to HIV-uninfected peers [125]. Perinatally infected adolescents have an increased risk of delayed puberty [134], which may impact their peak bone mass and their subsequent risk of osteoporosis and fractures [132,135]. To date, there are no studies investigating the rate of fractures among perinatally infected HIV patients [136]; long-term longitudinal studies are needed to fully assess this risk.

### Aetiology

Predictors of low BMD have been evaluated in several studies. Similar to adult studies [116,137–139], ART-treated HIV-infected adolescents appear to be at greater risk, with the use of protease inhibitors as a particular risk factor in some but not all studies [125,130,140]. The use of TDF has also been associated with low BMD in this population in some studies [35,139–142], likely due to decreased renal tubular phosphate reabsorption leading to hypophosphatemia and subsequent decreased bone mineralization [143]. However, this finding is not consistent among all studies, including a 60-month cohort study of 28 youth on TDF [144–146]. In adult studies, TDF is consistently associated with decreases in BMD in both ART switch studies and studies evaluating first-line regimens [46,138,147,148]. Most of the paediatric TDF studies have included a small number of subjects relative to adult studies, and thus, must be interpreted with caution. In Hazra *et al*. a younger age was associated with lower BMD, suggesting that this population may be at particular risk of TDF-related bone toxicity [141]. Full dose ritonavir alone or in combination with stavudine has also been associated with a low BMD in HIV-infected children and adolescents [149]. Additional HIV-related risk factors associated with low BMD vary by study and include advanced HIV stage, higher CD4 cell count, higher peak HIV-1 RNA levels and bone size [127,130,139,150] Traditional risk factors, as in adults, also contribute to lower BMD in HIV-infected adolescents, including lower weight and height Z-scores, white race and lack of multivitamin use [127,139].

### Management

The extent to which vitamin D deficiency contributes to low BMD in the HIV population is largely unknown, unlike in the general population where there are solid data from randomized, placebo-controlled trials that vitamin D and calcium supplementation decreases the risk of fractures and improves BMD in both adults and children [151–155]. In contrast, the studies that have been published within the HIV-infected population are mostly cross-sectional, observational, or retrospective in nature and show conflicting data. [156–161]. Only one study has been specifically designed to evaluate the bone effects of vitamin D supplementation in HIV-infected children and adolescents [162]. Arpadi *et al*. evaluated the bone mass accrual in 64 perinatally infected individuals, aged 6–16 years, after two years of 100,000 IU of vitamin D_3_ every other month plus daily calcium compared to placebo. No differences were found in bone mass parameters between the two groups after adjusting for confounding variables. However, while the intervention group increased their mean 25-hydroxyvitamin D (25(OH)D) concentrations after two years compared to the placebo group, 75% in the treatment group had at least 1 25(OH)D concentration <30 ng/mL, which is in the vitamin D insufficiency range. An important limitation of the study is that individuals with severe vitamin D deficiency (<12 ng/mL) were ineligible for the study, thus potentially excluding the group likely to benefit the most from the intervention. More data on bone disease among perinatally infected adolescents are needed to further characterize the prevalence of and risk factors associated with low BMD. In particular, more studies are needed to determine potential interventions that may minimize this population's long-term risk of osteoporosis and fractures. In the meantime, optimizing lifestyle choices, such as obtaining adequate nutrition and physical activity, and avoiding cigarette smoking, are crucial.

## Vitamin D deficiency

The prevalence of vitamin D deficiency, as measured by blood concentrations of 25-hydroxyvitamin D (25(OH)D), the established marker of overall vitamin D status [163] is very high in the HIV-infected population, including in HIV-infected adolescents [13,98,164–169]. In fact, in most studies the mean 25(OH)D values are well below current recommendations for both the Institute of Medicine (IOM) and The Endocrine Society [170,171]. A few studies have investigated risk factors for vitamin D deficiency in HIV-infected children and adolescents [13,165,166,168]. Non-HIV risk factors that have been identified include older age, female sex, black race, winter/spring season, higher BMI, and IR. Risk factors among HIV variables include longer duration of HIV disease and cumulative use of ART, NNRTIs, and NRTIs. Efavirenz and some PIs have been associated with vitamin D deficiency but their role in vivo is still unclear [172,173]. Havens *et al*. found an association between EFV use and baseline 25(OH)D concentrations; however, after three consecutive monthly vitamin D_3_ supplementation doses, EFV use did not attenuate the increase in 25(OH)D concentrations as observed in adult studies [169,174]. In contrast, Eckard *et al*. did not find an association with EFV use, but this was likely due to the majority of subjects having very low 25(OH)D concentrations. They did, however, find a strong association with Fitzpatrick skin type, which evaluates skin pigmentation, suggesting that this may be a better method of identifying people who are most at risk compared to using race [165]. More trials are needed to define the role that vitamin D plays on immune reconstitution and metabolic and cardiovascular co-morbidities, as well as the supplementation doses required to restore and maintain vitamin D sufficiency in HIV-infected children and adolescents.

## Conclusions

Metabolic complications of prolonged ART remain a serious and on-going problem of perinatally HIV-infected children, affecting their quality of life and long-term adherence to treatment. Longitudinal studies to document the incidence, risk factors and spectrum of disease in children are still limited. In resource-limited settings, these drug toxicities may progress unnoticed as large numbers of children initiate ART early in life and continue a lifetime of treatment with inadequate laboratory monitoring. Ethnic and lifestyle differences between children living in developed and resource-limited countries may have an impact on metabolic complications. Developing effective strategies to monitor, prevent and manage metabolic complications of ART in children and adolescents is critical. Therefore, using NRTIs with lower mitochondrial toxicity, simpler techniques for monitoring lipid profiles, identifying LD early, and promoting cardiac and bone health are priorities for improving long-term treatment outcomes.
